# WCCAN: Windowed Cross-contrast Attention Network for Multi-contrast Brain MR Image Super-resolution

**DOI:** 10.2463/mrms.mp.2026-0092

**Published:** 2026-07-25

**Authors:** Rania Saoudi, Djamel Eddine Boudechiche, Zoubeida Messali, Samir Brahim Belhaouari

**Affiliations:** 1 ETA Laboratory, IET Institute, University of Mohamed El Bachir El Ibrahimi, Bordj Bou Arreridj, Bordj Bou Arreridj, Algeria; 2 Department of Electrical Engineering and Automation, National Polytechnic School, Constantine, Constantine, Algeria; 3 Division of Information and Computer Technology, College of Science and Engineering, Hamad Bin Khalifa University, Doha, Qatar

**Keywords:** attention mechanisms, deep learning, magnetic resonance imaging, super-resolution, wavelet transforms

## Abstract

**Purpose:**

Most existing multi-contrast MRI super-resolution (MCMSR) methods rely on spatial-domain fusion and global attention, overlooking explicit high-frequency (HF) priors while incurring high computational costs. This work addresses these limitations through a general reference-guided MCMSR framework designed for low computational cost, applicable to any contrast pairing rather than a fixed clinical protocol.

**Methods:**

We introduce a wavelet-guided HF prior modeling block for directional-based decomposition and bounded nonlinear enhancement, enabling precise extraction and controlled amplification of anatomical details from both reference and target contrasts. We further introduce a triple cross-contrast fusion module, based on a windowed cross-contrast attention module, to efficiently transfer high-frequency information between contrasts and reduce computational complexity compared to global attention schemes. Additionally, to reduce feature differences across contrasts, a consistent feature fusion module with selective spatial adaptive modulation is incorporated.

**Results:**

Extensive experiments on IXI and M4Raw datasets demonstrate that our proposed windowed cross-contrast attention network (WCCAN) framework consistently outperforms state-of-the-art single and multi-contrast MRI SR methods in terms of quantitative accuracy and visual fidelity. In addition, the WCCAN model achieves lower computational complexity and faster inference time compared to the other MCMSR methods.

**Conclusion:**

The proposed WCCAN framework provides an efficient and accurate solution for MCMSR, demonstrating superior reconstruction quality with reduced computational cost compared to existing methods.

## Introduction

MRI is one of the most widely used imaging modalities for medical research. It employs a strong magnetic field combined with computerized radio waves to generate high-resolution (HR) images of human body organs, tissues, and cells without exposing patients to ionizing radiation, unlike other imaging techniques such as CT scans, X-ray imaging, and nuclear imaging.^1^^,^^2^ Due to its safety features, MRI technology has been widely used for the diagnosis of various diseases, such as brain tumors,^3^ breast cancers,^4^ cardiovascular disorders,^5^ musculoskeletal diseases,^6^ and renal abnormalities.^7^ However, acquiring MR images with an HR remains a critical challenge in practical applications due to hardware limitations and long scanning times, which may result in patient discomfort.^2^^,^^8^ To overcome these limitations, clinical MRI protocols typically acquire MRI scans using subsampled k-space strategies to accelerate the data acquisition. However, this aggressive reduction in acquired data can introduce motion artifacts and aliasing that significantly compromise image quality.^1^^,^^9^^,^^10^

Super-resolution (SR) techniques proposed in the literature^1^ have demonstrated a significant improvement in MR image resolution by recovering HR images from low-resolution (LR) inputs without requiring additional hardware updates. Conventional interpolation-based and sparse representation methods have limited capability to restore high-frequency (HF) anatomical details.^8^ More recently, deep learning–based SR approaches have demonstrated significant improvements in single-contrast MRI super-resolution (SCMSR), ranging from basic convolutional neural network (CNN) architectures^11^^–^^13^ to advanced generative models,^14^^–^^17^ attention-based frameworks,^18^^–^^20^ transformer-based architectures,^21^^,^^22^ and diffusion models.^23^^,^^24^ Although these approaches have achieved significant progress, they ignore the complementary information available in multiple MRI contrasts.

**Fig. 1:**
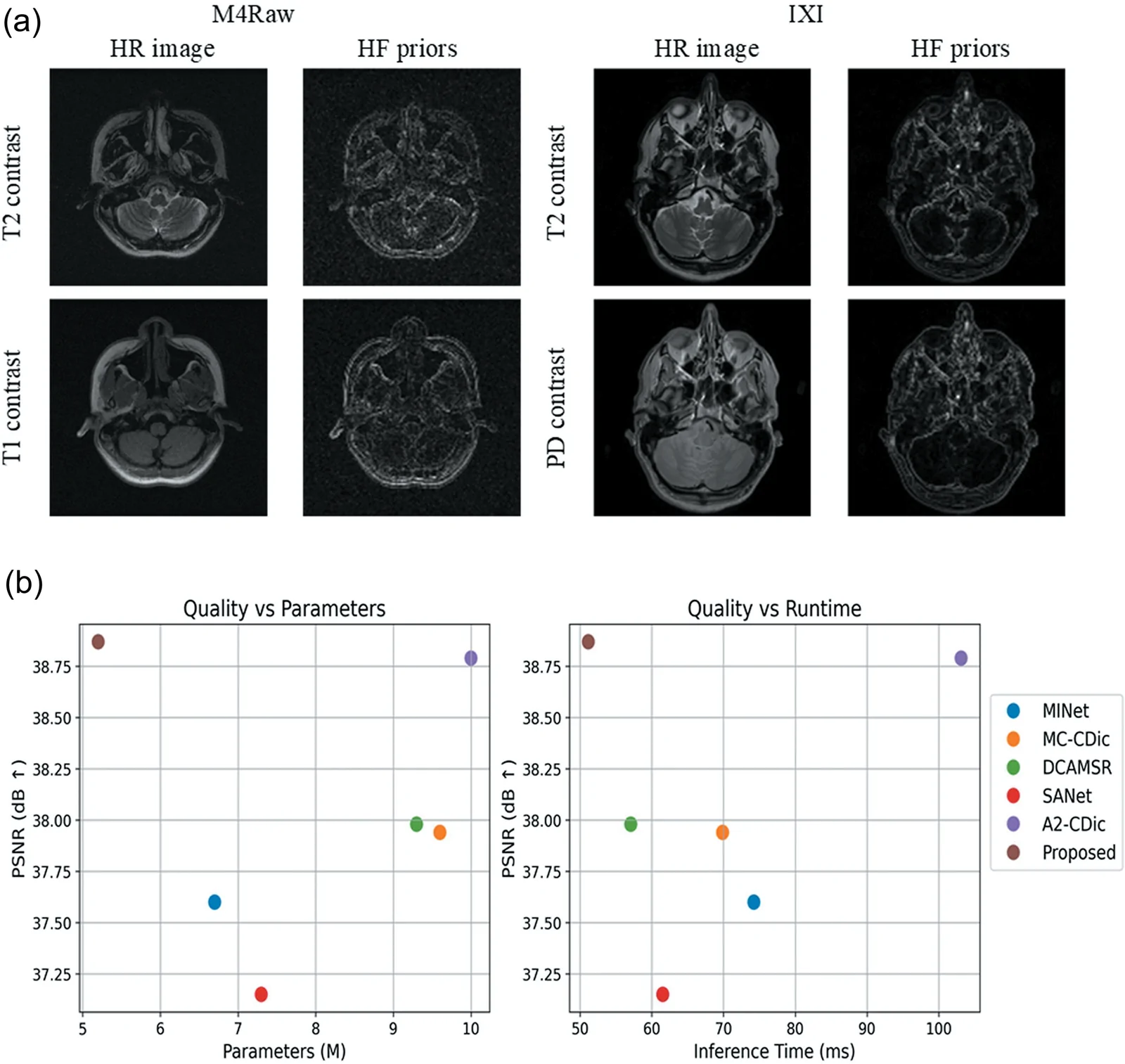
(**a**) Illustration of HR T2 with HR reference contrasts and their corresponding HF priors extracted via DWT operator. (**b**) Performance and complexity comparison of the proposed method against MCMSR approaches using IXI data with a scale factor of 4. A2-CDic, alignment-assisted multi-contrast convolutional dictionary; DCAMSR, dual cross-attention multi-contrast super-resolution; DWT, discrete wavelet transform; HF, high-frequency; HR, high-resolution; MCMSR, multi-contrast MRI super-resolution; MINet, multi-stage integration network; PSNR, peak signal-to-noise ratio; SANet, separable attention network; T1, T1-weighted; T2, T2-weighted.

In clinical practice, MRI protocols routinely generate multiple contrast images of the same anatomy by varying acquisition parameters such as TR and TE. Common contrasts include T1-weighted (T1), T2-weighted (T2), proton density–weighted (PD), fluid-attenuated inversion recovery (FLAIR), and fat-suppressed proton density–weighted (FS-PD) images, among others. Each contrast encodes distinct tissue properties: T1 highlights morphological and structural anatomy, T2 is sensitive to edema and inflammation, PD reflects soft tissue proton density, FLAIR suppresses cerebrospinal fluid signal to improve lesion conspicuity, and FS-PD suppresses fat signals to delineate cartilage and ligament structures.^1^ Despite differences in tissue contrast, images acquired from the same subject across different contrasts share remarkable similarities in HF anatomical details. As illustrated in Fig. 1a, the HR T2 and PD images from the IXI dataset,^25^ as well as the HR T2 and T1 images from the M4Raw dataset,^26^ acquired from the same subject, display distinct contrast while sharing consistent HF anatomical structure, such as edges and fine structures. This observation motivates the development of reference-guided multi-contrast MRI super-resolution (MCMSR) techniques, which exploit complementary structural information from HR reference contrasts to guide the reconstruction of HR target contrast images from their corresponding LR inputs, regardless of the specific contrast pairing available in a given protocol.^1^

However, effective cross-contrast fusion in MCMSR is challenged by spatial misalignment between reference and target images. These misalignments commonly arise from patient motion between sequentially acquired sequences; FOV and slice-prescription differences across contrasts; and sequence-dependent geometric distortions.^27^^,^^28^ Several fusion methods have been proposed to overcome these limitations, ranging from multi-stage integration and simple feature concatenation approaches^10^ to separable attention-based fusion models,^29^ multi-scale contextual matching frameworks,^30^ dual cross-attention transformer architectures,^31^ and integrated deformable convolutions with cross-attention transformer frameworks.^9^ Although these methods achieve improved reconstruction accuracy, they suffer from 3 critical limitations.

First, most existing approaches perform cross-contrast fusion primarily in the spatial domain,^9^^,^^10^^,^^29^^–^^31^ relying on implicit learning of anatomical structures without explicitly modeling HF information. Although some methods have integrated HF enhancement into their framework,^32^^,^^33^ this integration remains shallow and underutilized, limiting the precise recovery of complex anatomical details that are well preserved in fully sampled reference contrasts. Second, most attention-based fusion strategies rely on global attention mechanisms with quadratic computational complexity,^9^^,^^10^^,^^29^^,^^31^ resulting in high memory consumption and long inference time, which restricts their clinical applicability. Third, in MCMSR methods such as dual cross-attention multi-contrast super-resolution (DCAMSR),^31^ cross-contrast fusion uses standard spatial attention modulation (SAM) mechanisms^34^ without sufficient spatial selectivity, limiting their ability to address feature distribution mismatches caused by varying contrast intensities and misalignments, resulting in unstable cross-contrast integration.

To overcome these limitations, in this paper, we introduce a novel windowed cross-contrast attention network (WCCAN), which explicitly models directional HF anatomical priors from fully sampled reference contrasts and integrates them via a low-complexity cross-contrast fusion mechanism. Our main contributions are as follows:

• We propose a novel wavelet-guided high-frequency prior modeling block (WHPMB) that enables directional-based high-frequency decomposition and bounded nonlinear enhancement for precise recovery of anatomical edges and fine details from reference contrasts.
• We introduce a triple cross-contrast fusion module (TCCFM), which incorporates a novel windowed cross-contrast attention module (WCCAM) to facilitate efficient cross-window HF transfer with reduced computational complexities.
• We design a new consistent feature fusion module (CFFM) that uses a new selective spatial adaptive modulation (SSAM) block to reduce the feature differences across contrasts for accurate coherence.
• Extensive experimental results demonstrate that our framework outperforms existing state-of-the-art SCMSR and MCMSR methods in terms of quantitative metrics, visual quality, and computational efficiency.

## Materials and Methods

### Problem formulation and overall architecture

**Fig. 2:**
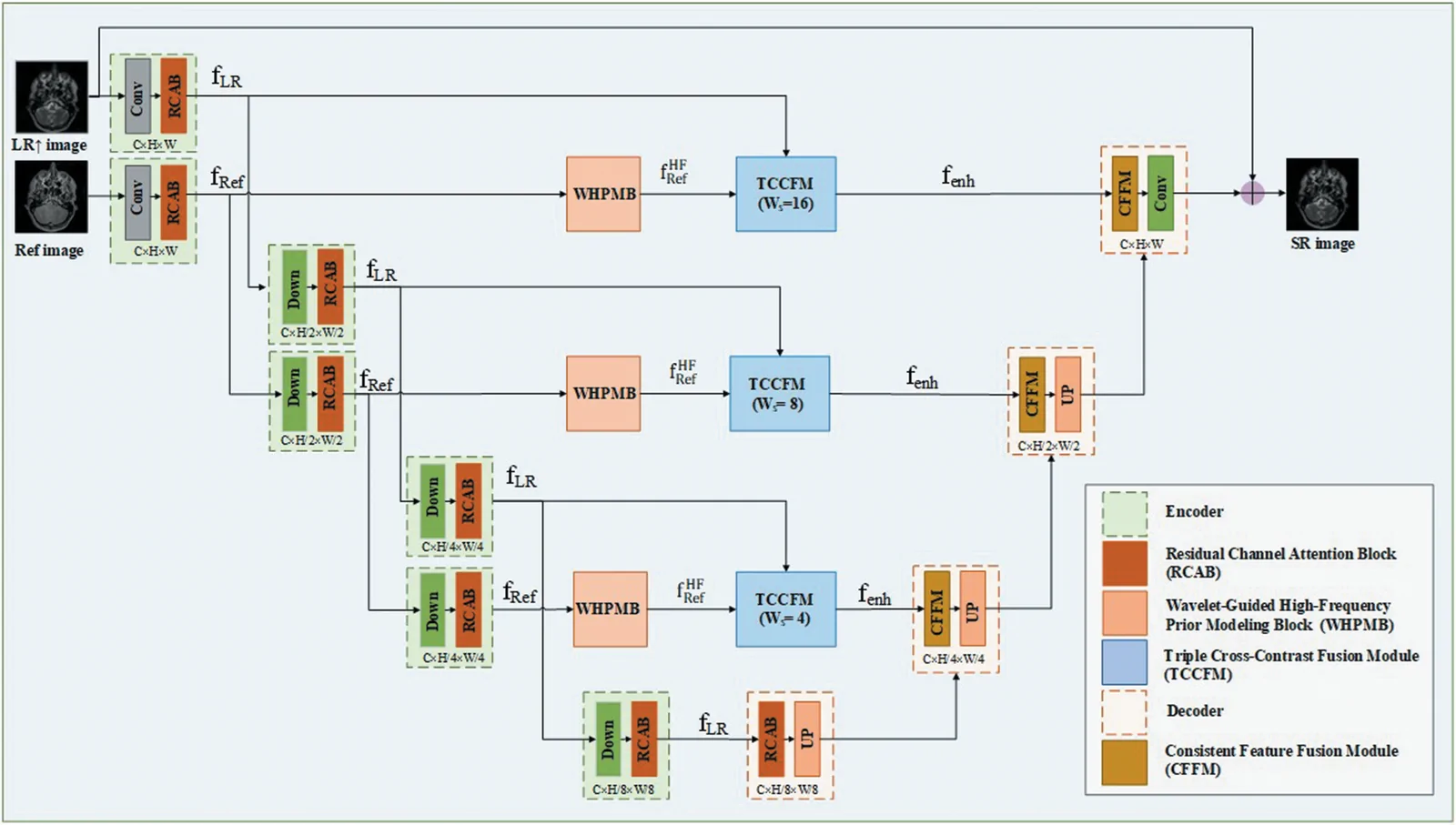
Overall architecture of our proposed WCCAN. The network employs dual encoders for initial feature extraction, a WHPMB block for HF prior enhancement, the TCCFM for effective cross-contrast feature fusion, and a decoder using CFFM for feature mismatch refinement and reconstruction. CFFM, consistent feature fusion module; Conv, convolutional layer; HF, high-frequency; LR, low-resolution; RCAB, residual channel attention blocks; Ref, reference; SR, super-resolution; TCCFM, triple cross-contrast fusion module; WCCAN, windowed cross-contrast attention network; WHPMB, wavelet-guided high-frequency prior modeling block.

The main objective of our study is to design a neural network that reconstructs a super-resolved image$\;I_{SR}$ ∈ R^H×W^ from a LR input $I_{LR}$ ∈ R^h×w^ using complementary information from a HR reference (Ref) image $I_{Ref}$ ∈ R^H×W^:

(1) $$I_{SR}=G\left(I_{LR},I_{Ref}\right),$$

where G (·,·) denotes the proposed WCCAN network. As illustrated in Fig. 2, WCCAN adopts a U-Net–based architecture consisting of dual encoders, a WHPMB, a TCCFM, and a hierarchical decoder. The LR input $I_{LR}\;$∈ R^h×w^ is first upsampled via bicubic interpolation to match the spatial resolution of the reference image $I_{Ref}\;$∈ R^H×W^. The upsampled LR image R^H×W^ and the HR reference image are initially processed through two parallel encoders, each composed of 3 levels, to extract initial multi-scale features $f_{LR}\;$and$\;f_{Ref}$, respectively.

At each encoder level, the proposed WHPMB explicitly enhances the reference features by amplifying their directional HF components, thereby producing enhanced HF representations$\;f_{Ref}^{HF}$. Subsequently, the $f_{Ref}^{HF}$ features are then integrated with $f_{LR}$ using the proposed TCCFM module to inject fine structural and textural details into the LR feature map through hierarchical cross-contrast fusion.

In the decoder, the fused features from TCCFM and the corresponding LR features are combined using the proposed CFFM and progressively upsampled to reconstruct the final HR-MRI output.

### Encoder

To effectively achieve cross-contrast alignment between the LR↑ inputs and HR reference images, we employ a dual-encoder U-Net–based architecture that is widely used for image feature alignment and segmentation.^31^^,^^35^^,^^36^ Each encoder consists of 3 hierarchical levels, enabling multi-scale feature extraction and mitigating or reducing misalignment between the inputs. At the first level, each input is passed through a shallow convolution layer, producing initial 64-channel feature maps, which are then refined using residual channel attention blocks (RCAB),^37^ resulting in $f_{LR}$ and $f_{Ref}$ ∈ *${\mathbb{R}}^{C\times H\times W}$^C×H×W^*. At the second and third levels, the features $f_{LR}$ and$\;f_{Ref}$ are progressively downsampled via a stride convolution layer and further refined with RCAB to construct rich hierarchical representations ($f_{Ref}$ at *R^C×H/2×W/2^* and *${\mathbb{R}}^{C\times H\times W}$^C×H/4×W/4^*, respectively). At level 4, only the LR branch is further refined using the RCAB to produce $\;f_{LR}$ ∈ R*^C×H/8×W/8^*.

### WHPMB

Prior MCMSR methods^32^^,^^33^ exploit the HF information from reference contrasts either by using wavelet priors only as auxiliary inputs without deep integration into the fusion process^32^ or implicitly learning the HF details without directional enhancement,^33^ limiting accurate modeling of anatomical edges and fine structures. To overcome these limitations, we propose a WHPMB, which performs learnable directional and bounded HF enhancement within a multi-scale framework.

**Fig. 3:**
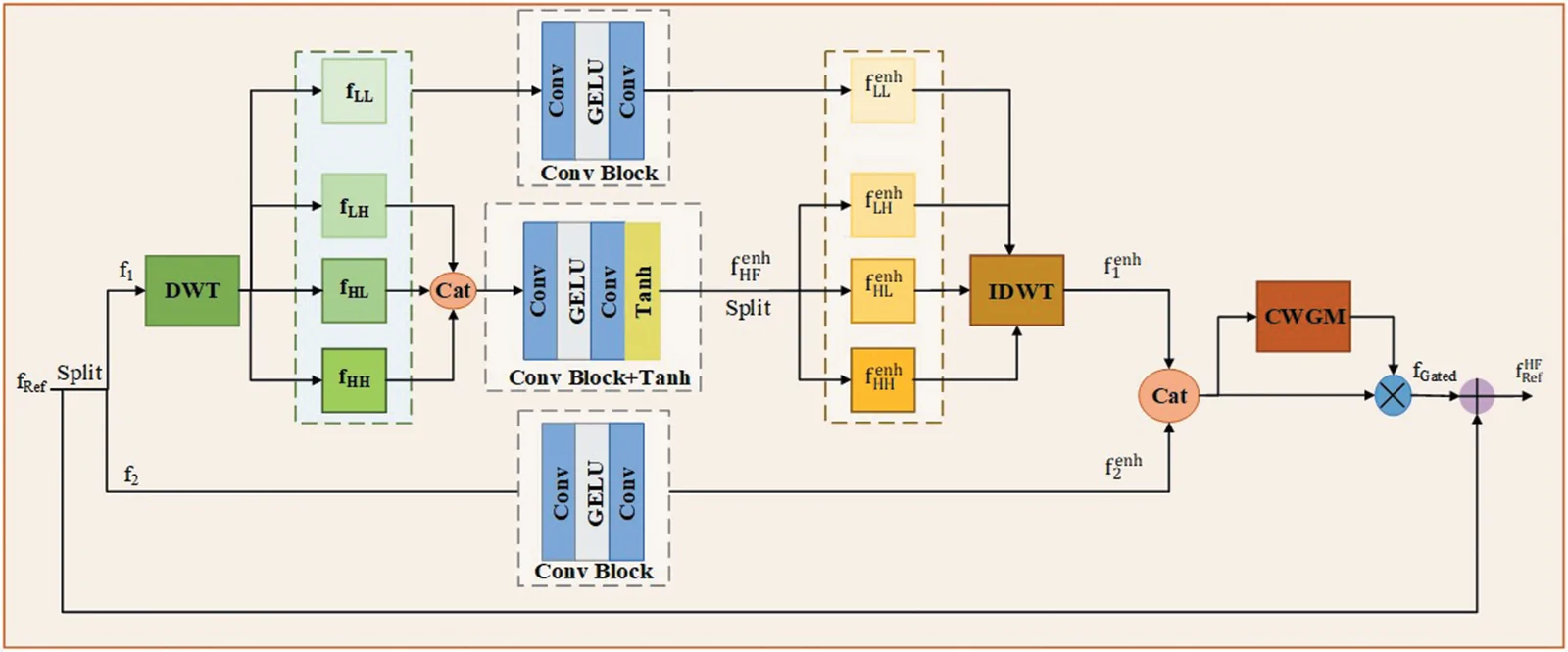
Proposed WHPMB architecture. The WHPMB actively enhances and amplifies directional HF components through DWT decomposition, bounded nonlinear enhancement, IDWT reconstruction, and final refinement via a CWGM mechanism. ConvBlock, convolutional block; CWGM, channel-wise gating mechanism; DWT, discrete wavelet transform; GELU, Gaussian Error Linear Unit; IDWT, inverse discrete wavelet transform; WHPMB, wavelet-guided high-frequency prior modeling block.

WHPMB uses the Haar discrete wavelet transform (DWT) method, a technique widely used for feature extraction in brain MRI disorder analysis.^38^^,^^39^ Aligning with our lightweight network design, we adopt a single-level 2D Haar wavelet due to its low computational complexity.^39^ As illustrated in Fig. 3, the proposed WHPMB first splits the input features $\;f_{1}$ and $\;f_{2}$.

In the first branch, $\;f_{LL}$ and 3 HF sub-bands. This decomposition separates the horizontal$\;f_{LH}$, vertical$\;f_{HH}\;$edge details, enabling directional HF enhancement from reference features. The low-frequency information $f_{LL}$ is refined using a lightweight convolutional block (ConvBlock), which comprises two 3 × 3 convolution layers and a Gaussian Error Linear Unit (GELU) activation function.^40^ In parallel, the concatenated HF coefficients [$f_{HL}$,$\;f_{HH}$] are first refined using ConvBlock and then processed by the nonlinear tanh activation function. We employ tanh activation to bound the enhanced HF coefficients within the range (−1, 1), consistent with prior image enhancement studies.^41^^,^^42^ Unlike unbounded activations, such as rectified linear unit (ReLU), tanh provides smooth nonlinear mapping and preserves gradient continuity for stable training. This is particularly important in MRI SR, where HF details are highly sensitive and any further enhancement may affect the anatomical structures and introduce artificial edges. The enhanced HF coefficients $f_{LH}^{enh}$, $f_{HL}^{enh}$, and $f_{LL}^{enh}$ via inverse DWT to reconstruct the enhanced $f_{1}^{enh}$ features.

In the second branch, $f_{2}^{enh}$. The enhanced features from both branches, $f_{1}^{enh\;}$and $f_{cat}$ and then fused through the channel-wise gating mechanism (CWGM),^43^ implemented using global average pooling, 2 fully connected layers, and sigmoid activation to extract the most relevant features from both branches. Finally, the gated features $\;f_{Gated}$ are added to the original $f_{Ref}^{HF}$. The detailed mathematical formulations of the proposed WHPMB are provided in supplementary equations (S1–S6).

### TCCFM

**Fig. 4:**
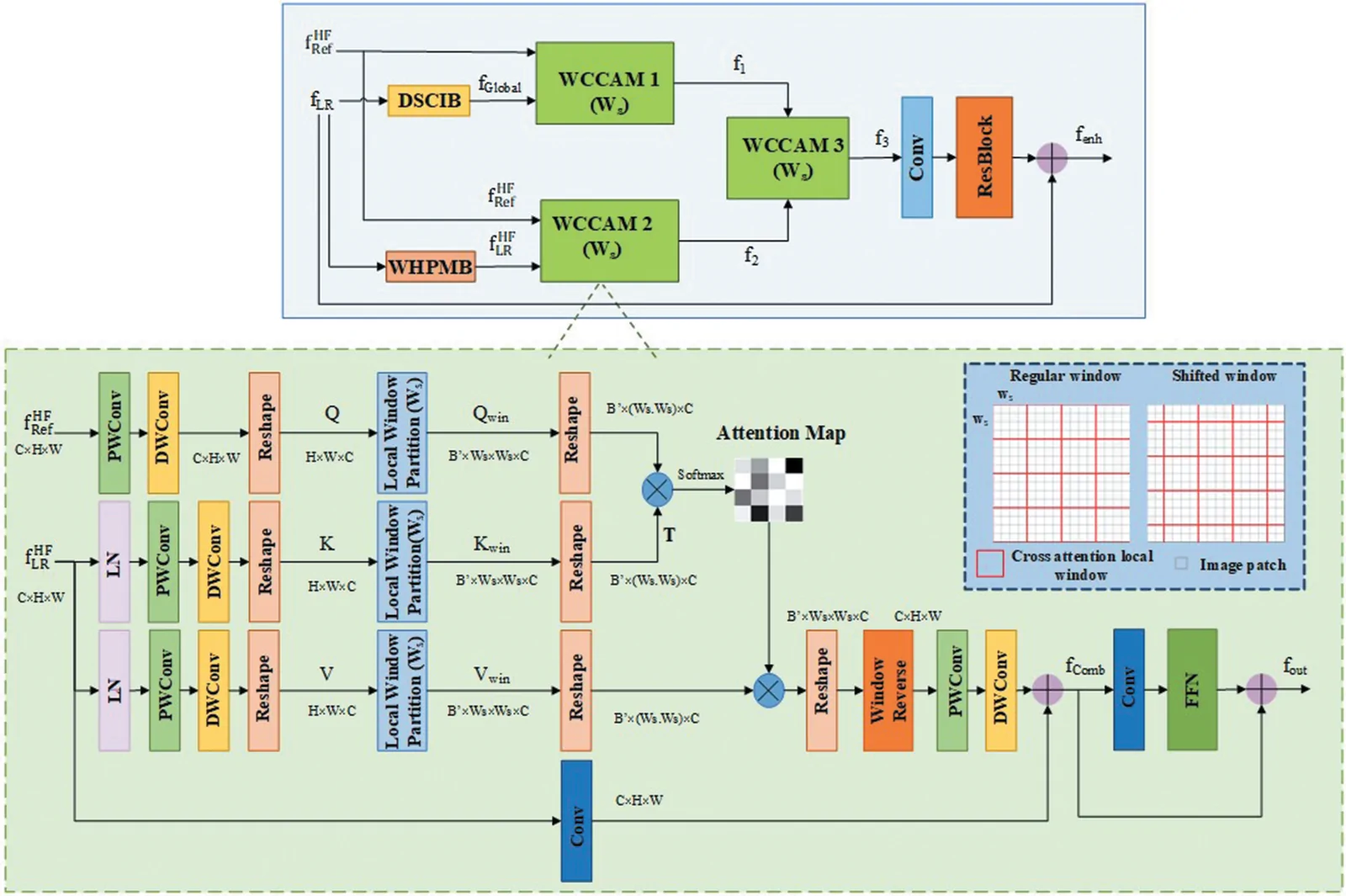
Proposed TCCFM with the WCCAM. TCCFM first employs DCSIB to extract global features from f_LR_ and WHPMB to amplify its HF details. Subsequently, it employs three WCCAMs to progressively integrate enhanced HF reference features, global target features, and enhanced HF target features for efficient cross-contrast information transfer. Conv, convolutional layer; DCSIB, dual-channel spatial interaction block; DW, depthwise; FFN, feed-forward network; HF, high-frequency; LN, layer normalization; PW, pointwise; TCCFM, triple cross-contrast fusion module; WCCAM, windowed cross-contrast attention module; WHPMB, wavelet-guided HF prior modeling block.

The TCCFM is a key component of the proposed WCCAN network. As illustrated in Fig. 4, TCCFM uses 2 inputs: the enhanced features $f_{Ref}^{HF}$ (obtained via the proposed WHPMB, as shown in Fig. 2) and the extracted features $f_{LR\;}$features are processed using a dual-path structure. Specifically, the first path employs a decoupled spatial-channel inverted bottleneck (DSCIB) block,^44^ which uses a decoupled linear and nonlinear mapping strategy to extract high global information $f_{Global\;}$from LR features. The second path uses the proposed WHPMB to amplify the HF components of $f_{LR}^{HF}$ feature maps. The enhanced features $f_{Ref}^{HF}$, $f_{LR}^{HF}$ are then fused through a 3-stage fusion scheme with parallel operation in the first 2 stages and hierarchical aggregation in the third stage.

TCCFM overcomes the limitations of existing multi-contrast fusion methods,^9^^,^^10^^,^^29^^,^^31^ by using the WCCAM that reduces high costs of global cross-attention with localized window-based interactions. Following the windowed attention strategy of the Swin Transformer,^45^ the WCCAMs alternate between regular and shifted windows. Specifically, the first stage employs a regular WCCAM between $f_{Global\;}\;$and $f_{LR}^{HF}\;$and $f_{Ref}^{HF}$. Unlike classical windowed cross-attention, this WCCAM focuses on enhanced HF components where each region in the enhanced LR features matches the most relevant corresponding features in the enhanced reference contrast, improving the edge sharpness and texture consistency. The third stage aggregates the outputs of the first 2 stages (denoted $f_{2}$) using regular WCCAM to balance the global context with precise HF restoration. The final fused representations $f_{3}$ are further refined with a 3 × 3 convolution and a residual block and then added to the input $f_{enh}$.

Inspired by the Swin Transformers^45^ and cross-view attention designs,^46^ we propose a windowed cross-contrast attention mechanism. As depicted in Fig. 4, the HF features of the reference image $f_{Ref}^{HF}\;$∈*ℝ^C×H×W^* are initially projected by a lightweight dual convolution projection (DCP) composed of a point-wise convolution (1 × 1), followed by a depth-wise convolution (3 × 3),^46^ generating query features Q∈ *R^C×H×W^*. In the parallel branch, the HF features of the target image R*^C×H×W^* are first normalized by Layer Normalization and then processed by the same DCP to generate both key and value features (K, R*^C×H×W^*).

To avoid the quadratic complexity of full global attention with (*HW × HW*) correlation maps, we adopt the local windowed attention with the (*w_s_ × w_s_*) strategy introduced in Liu et al,^45^ where the Q, R*^H×W×C^* and then partitioned into non-overlapping (*w_s_ × w_s_*) local windows. This partition generates windowed features {$Q_{win}$,$\;V_{win}$} ∈ *R^B’^*^*×Ws×Ws×C*^, where *B’* is the total number of windows, given by:

(2) $$B^{\prime }=\left(\frac{HW}{Ws^{2}}\right).$$

In the regular window configuration, partitioning begins at the top-left corner (0, 0), whereas in the shifted window configuration, a cyclic spatial shift of (*w_s_/2, w_s_/2*) is applied before window partitioning. Both windowing types are illustrated in Fig. 4. Each local window is reshaped into ℝ^B’× (Ws. Ws) ×C^, and the windowed cross-attention mechanism is computed.^45^^,^^47^ The detailed formulation of the windowed attention operation is presented in supplementary equation (S7).

Subsequently, the attended features are reshaped back to *R^B’×Ws×Ws×C^* and then returned to the original spatial size through a window reverse operation, resulting in feature maps of size *R^C×H×W^*. These outputs are further refined using DCP and added to the original $f_{Comb\;}$are then enhanced using a 3 × 3 convolution and feed-forward network (FFN). The FFN outputs are then added back to$\;f_{Comb\;}$for stable training and effective integration of the cross-contrast HF details, generating the final enhanced feature $f_{enh}$.

To balance global context extraction, HF detail enhancement, and computational efficiency, each TCCFM module in the proposed WCCAN network (Fig. 2) employs different window sizes. These window sizes are progressively decreased according to the spatial resolution of the reference and target HF feature maps, $f_{LR}^{HF}$ and $f_{Ref}^{HF}$. We use a window size of 16 × 16 at the highest resolution *R^C×H×W^*, 8 × 8 at the intermediate resolution *R^C×H/2×W/2^*, and 4 × 4 at the lowest resolution *R^C×H/4×W/4^*, respectively. We select these window sizes to maintain a consistent number of attention windows *B’* across scales, ensuring that the proposed WCCAM module enhances local HF details (such as edges and fine textures) at higher resolutions while capturing global structural consistency at lower resolutions. Furthermore, progressively decreasing the window size with feature resolution effectively reduces the quadratic attention complexity, leading to a significant reduction in memory usage and runtime.

### Decoder

In the decoder, we begin with the LR features at the fourth level, R*^C×H/8×W/8^*, which are first refined using an RCAB and then upsampled via a pixel shuffle layer to obtain intermediate features $f_{LR\;}$∈*R^C×H/4×W/4^*. These intermediate features are then fused with the enhanced TCCFM outputs $f_{enh\;}$∈R*^C×H/4×W/4^* through the proposed CFFM, producing the fused features R*^C×H/4×W/4^*. This process is repeated at the remaining levels, progressively upsampling the fused features and combining them with the corresponding enhanced features from the TCCFM $f_{enh\;}$at R*^C×H/2×W/2^* and R*^C×H×W^*, respectively. Finally, the fused features $f_{Fused\;}$are processed through a 3 × 3 convolution layer and added to the bicubic-upsampled LR input via a residual connection to produce the final HR-MRI output.

### CFFM

As the distribution of enhanced TCCFM features $f_{LR\;}$often differs, directly concatenating them and feeding them to standard convolution layers is not optimal for better alignment. Prior approaches^31^^,^^34^ addressed this issue using the SAM to remap the $f_{enh\;}$feature distribution with the LR domain through shallow affine predictors. However, these methods have limited representation capacity to model complex spatial–channel dependencies, which can lead to unstable feature integration.

**Fig. 5:**
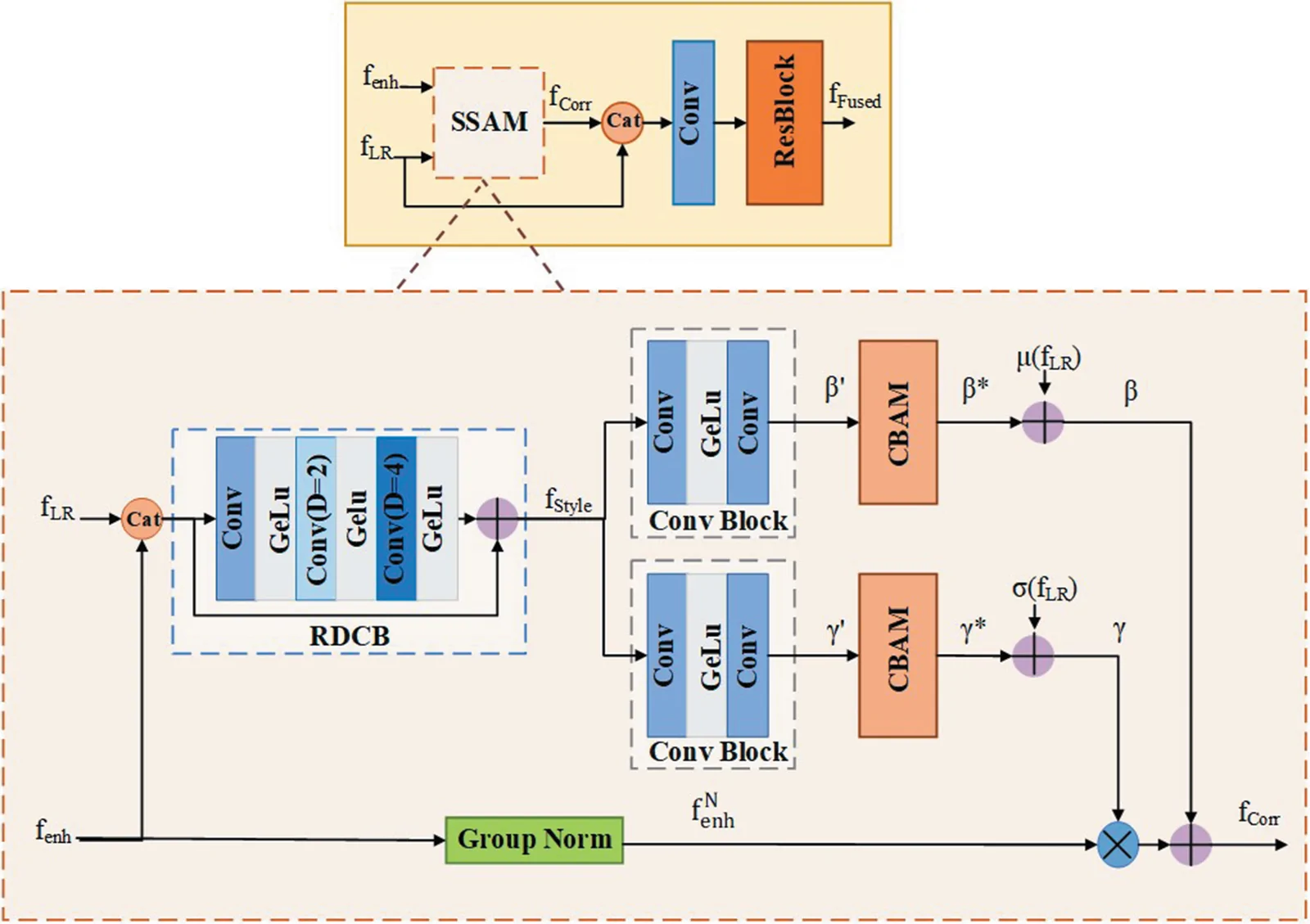
Overview of the proposed CFFM with a detailed architecture of the proposed SSAM that introduces selective spatial modulation to mitigate feature distribution mismatches. CBAM, convolutional block attention module; Conv, convolutional layer; CFFM, consistent feature fusion module; GeLu, Gaussian Error Linear Unit; RDCB, residual dilated convolutional block; ResBlock, residual block; SSAM, selective spatial adaptation module.

To overcome these limitations, we propose a CFFM that uses SSAM, which replaces shallow affine prediction with a deep, attention-guided modulation strategy for feature alignment and fusion. As illustrated in Fig. 5, SSAM first concatenates the *f_enh_* and $f_{cat\;}$features, and then operates via 2 parallel branches. In the first branch, the enhanced features $f_{enh}^{N}\;$to preserve the local contrast and stabilize training under small batch sizes. In the second branch, the $f_{Style}$. The extracted style features ^48^ to enable channel-wise selection and spatially adaptive modulation, producing refined γ* and β* parameters.

Subsequently, we inject the mean and variance of the $f_{enh\;}$and the $f_{LR\;}$feature domains as follows:

(3) $$\gamma =\gamma^{*}+\mu (f_{LR})$$

(4) $$\beta =\beta^{*}+\sigma (f_{LR}).$$

Subsequently, we use the adjusted parameters *γ* and *β* to modulate the normalized enhanced features$\;f_{enh}^{N}$, producing the corrected features:

(5) $$f_{corr}=\gamma ⊙\;f_{enh}^{N}+\beta ,$$

where ⊙ denotes element-wise multiplication. The corrected features are then concatenated with the input features $f_{Fused}$.

### Datasets

#### Acquisition details

We evaluated our proposed model using 2 publicly available multi-contrast brain MRI datasets, IXI^25^ and M4Raw,^26^ selected as complementary benchmarks for evaluating the generalizability of our proposed framework across different contrast pairings and acquisition settings. The IXI dataset comprises T1, T2, and PD brain MR images acquired on 3 scanners: a Philips Intera 3T (Philips Healthcare, Amsterdam, the Netherlands; Hammersmith Hospital), a Philips Gyroscan Intera 1.5T (Philips Healthcare; Guy’s Hospital), and a GE Signa 1.5T (GE Healthcare, Chicago, IL, USA; Institute of Psychiatry). The datasets are provided as 3D volumes with a voxel size of approximately 0.9375 × 0.9375 × 1.25 mm^3^. The M4Raw dataset provides a multi-channel MRI k-space dataset for low-field MRI research, acquired on a 0.3 Tesla whole-body MRI system (OPER-0.3; Ningbo Xingaoyi, Zhejiang, China) with a 4-channel head coil. It consists of 2D multi-slice acquisitions that include T1, T2, and FLAIR contrasts, each acquiring 18 slices with a thickness of 5 mm and an in-plane resolution of 0.94 × 1.23 mm^2^. Both T1 and T2 datasets were acquired with 3 individual repetitions, while FLAIR was acquired with 2 repetitions. Further acquisition parameters for both datasets are provided in Table 1.

#### Preprocessing

All datasets were preprocessed using the same pipeline across all compared methods. For the IXI datasets, we used paired T2 and PD contrasts. For each contrast pair, we selected 160 subjects for training and 16 subjects for testing. The datasets are provided as 3D volumes stored in NIfTI format. From each volume, we extracted 2D axial slices to train our network. Slices in which non-zero voxels accounted for less than 10% of the total were discarded, resulting in 20000 images for training and 2000 images for testing.

For the M4Raw datasets, we used paired T2 and T1 contrasts. For each contrast pair, we selected 128 subjects for training and 30 subjects for testing, all stored in HDF5 format. Since each contrast was acquired with 3 repetitions, this corresponds to 384 volumes for training and 90 volumes for testing per contrast, respectively. We follow the same preprocessing strategy and generate 6912 training images and 1620 testing images similar to prior work.^31^ In both datasets, the multi-contrast images are acquired from the same subject within the same session and scanner; therefore, no additional registration step was applied. All images are independently normalized to the range [0, 1].

#### LR image generation

The original HR images in both datasets are 256 × 256 pixels. Following the standard k-space downsampling strategy used in existing MCMSR methods,^10^^,^^27^^,^^29^^,^^31^^,^^33^ first we applied a 2D fast Fourier transform (FFT) to the HR image to obtain its full k-space representation. Second, we cropped only the central low-frequency region and removed HF components. For ×2 downsampling, we retained the central (1/2 × 1/2) k-space region corresponding to 25% of the total k-space samples, whereas for ×4 downsampling, we retained the central (1/4 × 1/4) k-space region corresponding to 6.25% of the samples. Finally, we reconstructed the spatial-domain LR images by performing an inverse 2D FFT directly on the cropped k-space.

### Implementation details

We implemented the proposed WCCAN framework using the PyTorch framework (Meta AI, Menlo Park, CA, USA) and trained it on an NVIDIA RTX 4090 GPU (NVIDIA, Santa Clara, CA, USA) for 50 epochs, with a batch size of 4. For the optimization process, we used the Adam optimizer with a learning rate of 0.0002, β_1_ = 0.9, and β_2_ = 0.999 to minimize the standard pixel-wise L1 loss between SR and HR images.

### Model comparison details

We evaluated the effectiveness of our WCCAN model against 9 advanced SCMSR and MCMSR methods. For a fair comparison with the SCMSR methods, we evaluated our WCCAN baseline with 4 architectures specifically designed for MRI enhancement: magnetic resonance dense network (MRDN),^11^ super-resolution DenseNet (SRDenseNet),^12^ channel splitting network (CSN),^18^ and enhanced deep super-resolution network with multi-head convolutional attention (EDSR + MHCA).^19^ Furthermore, we compared the full WCCAN model with MC MSR methods including, multi-stage integration network (MINet),^10^ multi-contrast convolutional dictionary (MC-CDic),^8^ DCAMSR,^31^ separable attention network (SANet),^29^ and alignment-assisted multi-contrast convolutional dictionary (A2-CDic)^27^ models. All models were retrained from scratch following their official training configurations using the same training and testing splits on the IXI and M4Raw datasets and the same frequency-domain downsampling process.

To analyze the complexity of the proposed WCCAN, we performed a detailed comparison with other MCMSR methods in terms of parameter count, floating-point operations (FLOPs), peak GPU memory consumption, and runtime. All measurements were performed under identical conditions, with a 256 × 256 input resolution and a ×4 upscaling factor.

### Evaluation metrics

We employed common pixel similarity metrics used for MRI evaluation^49^ to evaluate the proposed WCCAN model, including peak SNR (PSNR), structural similarity index measure (SSIM), root mean squared error (RMSE), high-frequency error norm (HFEN),^50^ and learned perceptual image patch similarity (LPIPS). Higher PSNR and SSIM values along with lower RMSE, HFEN, and LPIPS values indicate better performance. In addition, we qualitatively evaluated the SR images using visual quality.

### Ablation study design

To evaluate the contribution of each component in WCCAN, 2 ablation studies were conducted.

The first is a progressive ablation in which modules are added incrementally to a baseline model. The baseline model processes only the LR T2 image as input, without any reference contrast or cross-contrast interaction.

The second is a component-level ablation in which one component is varied while the others are held fixed. For the WHPMB module, we retain TCCFM and SSAM and modify the HF enhancement strategy in the reference branch. We compare 3 variants: (1) without HF enhancement; (2) non-directional HF enhancement; and (3) the full WHPMB with Haar-based directional HF enhancement. For the TCCFM module, we fix WHPMB and SSAM and evaluate 2 fusion strategies: (1) a simple channel concatenation of $f_{Ref}^{HF}$ features and (2) fusion using the proposed TCCFM. For the SSAM module, we retain WHPM B and TCCFM and compare 3 integration strategies: (1) direct feature concatenation; (2) SAM-based fusion^31^^,^^34^; and (3) the proposed SSAM.

All configurations in both ablation studies are trained and evaluated under the same conditions, dataset splits, preprocessing, k-space downsampling, optimizer, learning rate schedule, and number of epochs so that observed performance differences can be attributed directly to the component under evaluation.

## Results

### Quantitative and qualitative evaluation

As shown in Tables 2 and 3, the full WCCAN model achieves the highest PSNR and SSIM along with the lowest RMSE and HFEN values across both datasets and both scaling factors, outperforming all compared SCMSR and MCMSR methods.

**Fig. 6:**
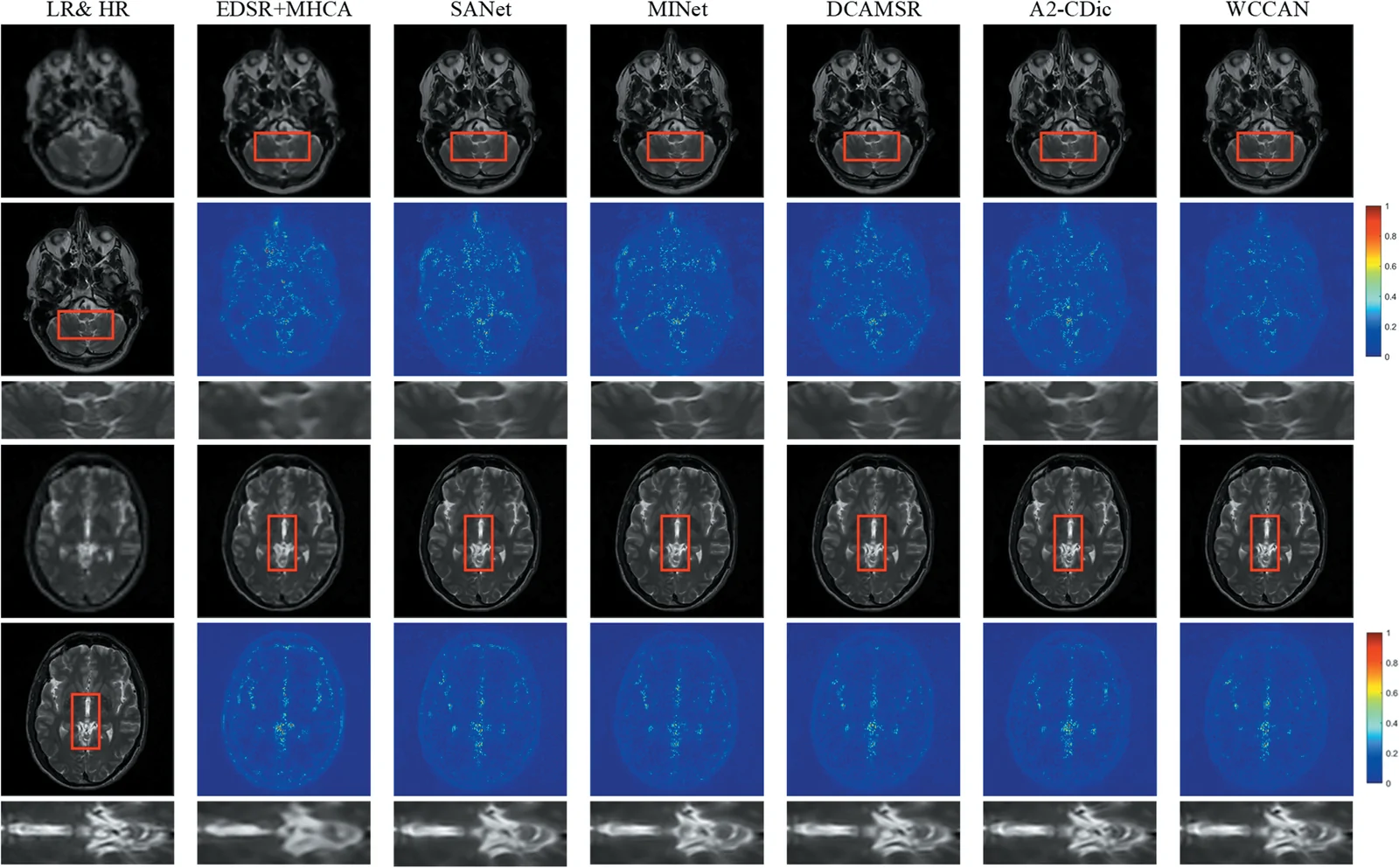
Qualitative results and error maps of different SR methods on IXI data under a scale factor of 4. From top to bottom: reconstructed results of competing methods, corresponding error maps, and zoomed-in regions highlighted by red rectangles to facilitate visual comparison of fine details. Brighter error maps indicate more reconstruction errors. A2-CDic, alignment-assisted multi-contrast convolutional dictionary; DCAMSR, dual cross-attention multi-contrast super-resolution; EDSR + MHCA, enhanced deep super-resolution network with multi-head convolutional attention; HR, high-resolution; LR, low-resolution; MINet, multi-stage integration network; SANet, separable attention network; SR, super-resolution; WCCAN, windowed cross-contrast attention network.

**Fig. 7:**
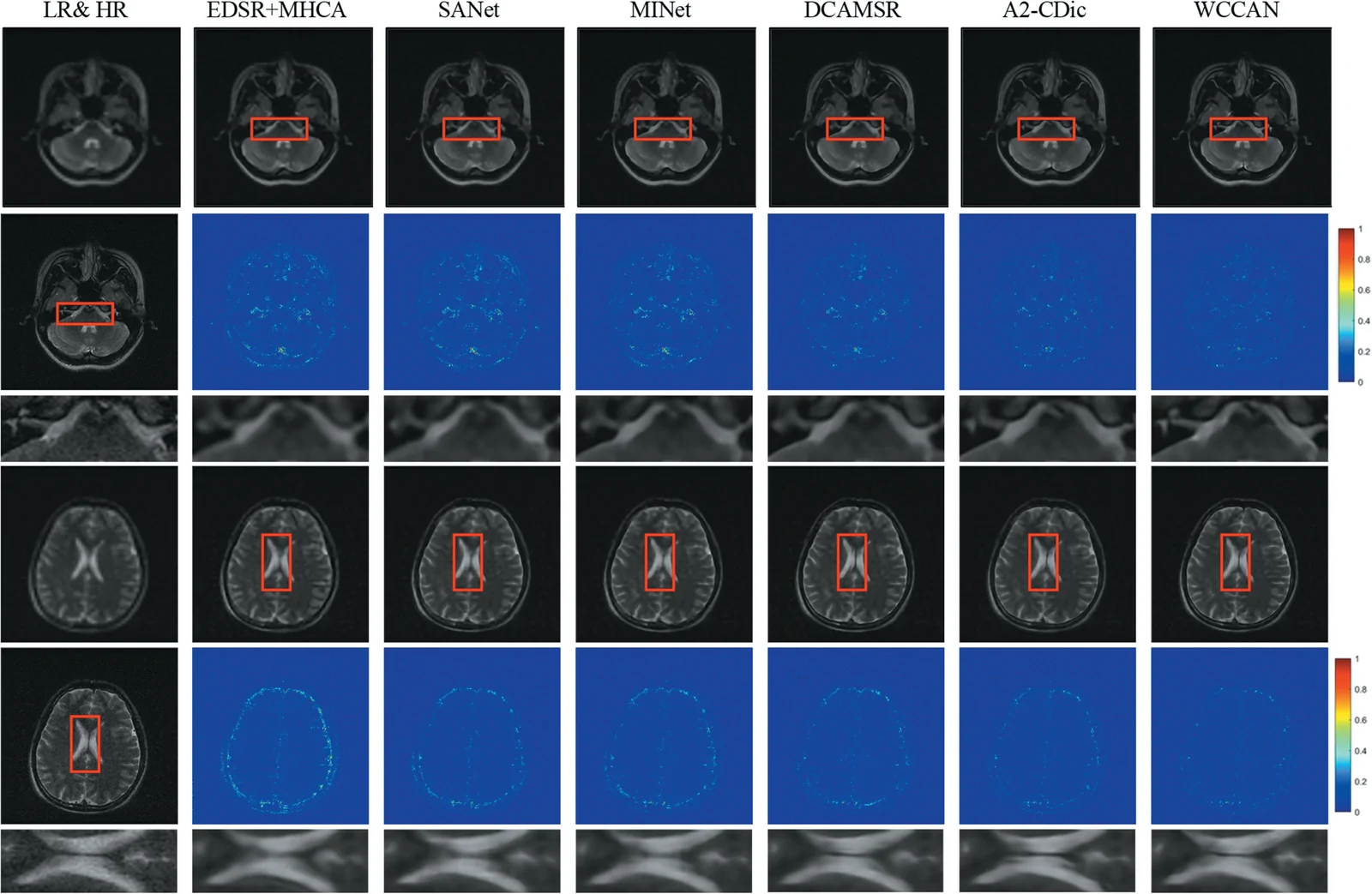
Qualitative results and error maps of different SR methods on M4Raw data under a scale factor of 4. From top to bottom: reconstructed results of competing methods, corresponding error maps, and enlarged views of the regions indicated by red rectangles for clearer visualization. Brighter error maps indicate more reconstruction errors. A2-CDic, alignment-assisted multi-contrast convolutional dictionary; DCAMSR, dual cross-attention multi-contrast super-resolution; EDSR + MHCA, enhanced deep super-resolution network with multi-head convolutional attention; HR, high-resolution; LR, low-resolution; MINet, multi-stage integration network; SANet, separable attention network; SR, super-resolution; WCCAN, windowed cross-contrast attention network.

Figures 6 and 7 present visual comparisons of the reconstructed images and their corresponding error maps from various SR networks for a ×4 scaling factor on the IXI and M4Raw datasets, respectively. Zoomed views of the regions highlighted by red rectangles are included to better illustrate fine structural recovery. On the IXI dataset (Fig. 6), the WCCAN model clearly outperformed all competing SR methods, providing improved images with well-defined edges and textures and a significant reduction in blurring artifacts. This is further reflected in the error maps, where the proposed WCCAN method exhibits a lower residual error than other competing methods. The same pattern holds on the M4Raw dataset (Fig. 7), where WCCAN recovers sharper edges and finer structures with lower residual error than all compared methods.

### Complexity analysis

As shown in Table 4, among all compared MCMSR methods, WCCAN requires the fewest parameters (5.2 meter), lowest FLOPs (125.45 G), smallest peak GPU memory (2.97 GB), and shortest inference time (51.16 ms). Fig. 1b further illustrates the position of WCCAN relative to other MCMSR methods in the accuracy-complexity trade-off space, showing that WCCAN achieves high reconstruction accuracy at lower computational cost than all compared methods.

### Ablation study results

Table 5 presents the results of the progressive ablation study on the IXI and M4Raw datasets at a ×4 scaling factor. Performance improves consistently as each proposed module is added to the baseline. Introducing the reference contrast via the proposed WHPMB and applying directional HF prior modeling provides the largest single-step improvement, increasing PSNR by +2.74 dB on IXI and +0.36 dB on M4Raw, with notable improvements in SSIM, HFEN, and LPIPS. Further incorporating TCCFM produces additional gains in HF recovery, where HFEN decreases from 1.0022 to 0.7834 on IXI and from 1.3829 to 1.3261 on M4Raw. Finally, adding SSAM yields the best overall performance, achieving the highest PSNR and SSIM with the lowest HFEN and LPIPS values on both datasets.

**Fig. 8:**
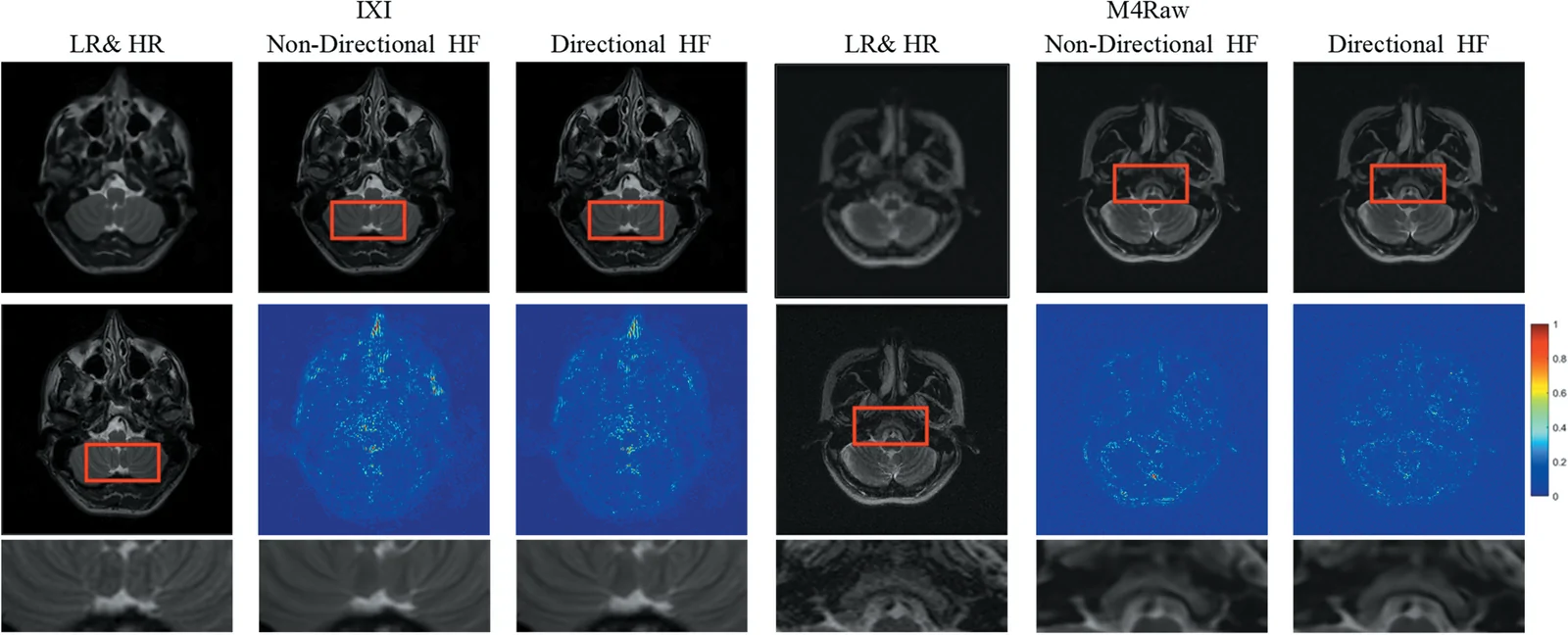
Qualitative comparisons and error maps between non-directional and directional HF enhancement on both IXI and M4Raw datasets under a ×4 upscaling factor. Red rectangles indicate the regions enlarged in the zoomed-in views for detailed comparison. HF, high-frequency; HR, high-resolution; LR, low-resolution.

Table 6 presents the results of the component-level ablation, in which each proposed module is varied individually while the others are held fixed. For WHPMB, removing it entirely causes clear degradation across all metrics. Introducing WHPMB without directional HF extraction already improves reconstruction quality, PSNR increases by 1.12 dB on IXI and 0.37 dB on M4Raw, and HFEN decreases from 0.8913 to 0.7291 on IXI and from 1.3999 to 1.2874 on M4Raw. The full WHPMB with directional HF enhancement achieves the best results across all metrics, as reflected in the sharpest and most well-defined structures in Fig. 8. Additionally, the directional variant slightly reduces the parameter count to 5.2 metre compared to the non-directional variant.

For TCCFM, simple channel concatenation of the LR and enhanced HF features produces lower PSNR and SSIM and higher HFEN and LPIPS than the proposed module. Replacing concatenation with TCCFM increases PSNR by 1.2 dB on IXI and 0.36 dB on M4Raw, with consistent improvements in SSIM, HFEN, and LPIPS, at a parameter cost of 1.2 metre.

For SSAM, direct feature concatenation results in limited interaction between the LR and enhanced features, leading to lower HF recovery and higher perceptual error. SAM-based fusion^31^^,^^34^ improves spatial focus and perceptual quality, increasing PSNR by 0.7 dB on IXI and reducing LPIPS by 0.0016 on IXI, but performance remains below the proposed SSAM. SSAM achieves the highest PSNR and SSIM with the lowest HFEN and LPIPS on both IXI and M4Raw datasets at a parameter count of 5.2 metre.

## Discussion

SCMSR methods consistently produced the lowest PSNR and SSIM and the highest RMSE and HFEN values across both datasets and both scale factors. This confirms that recovering fine anatomical detail from a single degraded input is fundamentally limited and the missing HF information cannot be inferred from the target contrast alone when the degradation is severe, particularly at ×4 scaling.

MCMSR methods that incorporate reference contrasts, MINet^10^ and SANet,^29^ show clear improvements over SCMSR methods across all metrics. However, their multi-stage integration and separable attention strategies fail to preserve fine structural details at ×4 scaling, where the degradation removes most recoverable HF content. Methods that rely on global cross-attention, MC-CDi,^29^ DCAMSR,^31^ and A2-CDic,^27^ achieve more competitive PSNR and SSIM but still show elevated RMSE and HFEN values relative to WCCAN. None of these methods explicitly model HF structural priors from the reference contrast. Cross-contrast feature transfer in the spatial domain can capture global structural correspondence but cannot reliably recover directional edge and texture detail, which requires explicit frequency-domain modeling. These methods also incur substantially higher computational cost, parameter counts, FLOPs, peak memory, and inference time all exceed those of WCCAN, as shown in Table 4.

The ablation results directly attribute WCCAN’s performance gains to its 3 proposed components. Adding WHPMB produces the largest single-step improvement in the progressive ablation, +2.74 dB PSNR on IXI and +0.36 dB on M4Raw, confirming that explicit directional HF prior modeling from the reference contrast is the primary driver of reconstruction quality. The component-level ablation further shows that removing directional HF extraction from WHPMB degrades HFEN from 0.7291 to 0.8913 on IXI and from 1.2874 to 1.3999 on M4Raw, while the non-directional variant still underperforms the full WHPMB. This confirms that the directional decomposition into the high-low frequency (HL), low-high frequency (LH), and high-high frequency (HH) sub-bands, rather than HF modeling alone, is what enables precise anatomical edge recovery. The mechanism is the bounded nonlinear enhancement applied separately to each directional sub-band, which amplifies contrast-specific structural detail without the instability that unbounded activation would introduce.

Adding TCCFM produces the largest improvement in HFEN, from 1.0022 to 0.7834 on IXI and from 1.3829 to 1.3261 on M4Raw, which reflects more accurate recovery of fine anatomical structures through cross-contrast feature interaction. By restricting cross-contrast attention to shifted local windows, TCCFM transfers HF structural detail between contrasts more precisely than global attention, which tends to distribute attention over spatially distant and often irrelevant regions. The alternating regular and shifted window structure additionally captures longer-range cross-contrast correspondence that a single window configuration would miss, contributing to the consistent improvements in both HFEN and PSNR.

Adding SSAM produces the largest improvement in LPIPS, the perceptual quality metric, on both datasets, confirming that the feature distribution mismatch between the TCCFM outputs and the LR target features is a meaningful source of perceptual artifacts. The component-level ablation shows that SAM-based fusion^31^^,^^34^ partially addresses this, it increases PSNR by 0.7 dB on IXI and reduces LPIPS by 0.0016, but performance remains below SSAM. The limitation of SAM is its shallow affine predictor, which applies a uniform global scale and shift that cannot capture the spatially varying contrast-intensity differences between T2 and PD (or T1) images. SSAM’s joint conditioning on both the TCCFM-enhanced features and the LR target features enables spatially selective suppression of contrast-inconsistent regions, producing the highest PSNR and SSIM with the lowest HFEN and LPIPS across both datasets.

The model is trained using L1 loss. Despite the absence of perceptual or adversarial loss terms, WCCAN achieves the best HFEN and LPIPS values across all compared methods. This confirms that the architectural design, explicit HF prior modeling, windowed cross-contrast fusion, and selective spatial modulation are sufficient to recover fine anatomical detail and suppress perceptual artifacts. Perceptual losses are known to introduce hallucinated HF structures in MRI SR,^51^ which is clinically undesirable. L1 avoids this while producing sharper edges and better structural fidelity than L2^52^^,^^53^ due to its lower sensitivity to intensity outliers and resistance to over-smoothing.

WCCAN is designed to operate on any pair of simultaneously acquired contrasts: T1-guided T2 enhancement, FLAIR-guided T2 reconstruction, or other pairings depending on the local protocol. The model operates on reconstructed magnitude images, consistent with how MR images are stored and accessed in clinical practice via DICOM and PACS.^54^ It can therefore be integrated as a post-processing step before radiological review without modifying the acquisition protocol or requiring access to raw k-space data.

### Limitations

The contrast pairings evaluated, T2/PD on IXI and T2/T1 on M4Raw, reflect benchmark availability rather than current clinical standards, where FLAIR has largely replaced PD in standard neurological brain MRI. FLAIR is not available in IXI but is included in M4Raw alongside T1 and T2. Evaluating WCCAN on FLAIR-guided SR is a direct next step and would strengthen the clinical relevance of the framework.

WCCAN is a 2D slice-based framework that processes each axial slice independently without explicitly enforcing consistency across adjacent slices. As a result, minor through-plane intensity inconsistencies may appear when reconstructed slices are assembled into a 3D volume. This limitation is shared by all compared methods and could be addressed in future work through a fully 3D volumetric formulation.

LR images are generated by central k-space cropping, retaining 25% of samples for ×2 and 6.25% for ×4 scaling, which does not replicate the variable-density undersampling, partial Fourier acquisition, or parallel imaging strategies used in clinical MRI acceleration. Performance under clinically realistic undersampling conditions has not been evaluated. The raw k-space data in M4Raw provides a concrete pathway for this evaluation in future work.

WCCAN operates on magnitude images and does not incorporate raw multi-coil k-space information or phase data. This enables DICOM-compatible clinical deployment but discards coil-sensitivity diversity and phase information that reconstruction-domain approaches can exploit.

The robustness of WCCAN to inter-contrast misregistration has not been quantitatively evaluated. While both datasets provide approximately aligned multi-contrast images by acquisition design, all contrasts are acquired within the same session on the same scanner; residual misalignment from patient motion between sequences cannot be excluded. Systematic robustness evaluation and the potential benefit of integrating a learnable registration module remain open questions.

## Conclusion

In this paper, we propose WCCAN, a general reference-guided framework for cross-contrast MRI SR, applicable to any contrast pairing available in a given clinical setting rather than tied to a specific acquisition protocol. Unlike existing methods that rely on spatial-domain fusion and global attention mechanisms, which overlook explicit HF priors and incur prohibitive computational costs, WCCAN addresses these limitations through 3 complementary components: a WHPMB for directional decomposition and bounded nonlinear enhancement of anatomical details from reference contrasts; a TCCFM incorporating a WCCAM for efficient cross-contrast HF transfer with linear complexity; and a CFFM with SSAM for stable and accurate cross-contrast integration. Ablation studies confirm that each component contributes independently, WHPMB drives HF detail recovery, WCCAM enables efficient cross-contrast feature transfer, and SSAM reduces perceptual artifacts from cross-contrast feature distribution mismatches. Extensive evaluations on the IXI and M4Raw datasets demonstrate that WCCAN achieves the highest PSNR, SSIM, HFEN, and RMSE scores across all compared SCMSR and MCMSR methods while maintaining lower computational complexity and faster inference time, making it a practical solution for high-quality MRI SR in clinical workflows.

## Figures and Tables

**Table 1:** Acquisition parameters of the IXI and M4Raw datasets

Scan parameters	IXI (Guy’s Hospital)		M4Raw	
	T2 images	PD images	T2 images	T1 images
Scanner	Philips Gyroscan Intera	Philips Gyroscan Intera	OPER-0.3, Ningbo Xingaoyi	OPER-0.3, Ningbo Xingaoyi
Field strength	1.5T	1.5T	0.3T	0.3T
TR (ms)	8178.34	8178.34	5500	500
TE (ms)	100	8	128	18.4

PD, proton density–weighted.

**Table 2:** Quantitative results of different SCMSR and MCMSR methods on IXI datasets under ×2 and ×4 scale factors

SR methods	Params	2×				4×			
		PSNR↑	SSIM↑	RMSE↓	HFEN↓	PSNR↑	SSIM↑	RMSE↓	HFEN↓
CSN^18^ (2019)	10.6 M	40.66	0.9834	2.6422	0.2440	32.79	0.9150	6.6130	1.5187
SRDenseNet^12^ (2022)	6.6 M	40.60	0.9831	2.6629	0.2527	32.84	0.9207	6.6028	1.5181
MRDN^11^ (2021)	8.7 M	40.79	0.9836	2.6036	0.2423	32.83	0.9210	6.5906	1.5089
EDSR + MHCA^19^ (2023)	**2.5 M**	40.82	0.9837	2.5975	0.2414	32.93	0.9222	6.5507	1.5033
**WCCAN (Baseline)**	3.5 M	**41.09**	**0.9845**	**2.5015**	**0.2412**	**33.35**	**0.9278**	**6.1765**	**1.4032**
MINet^10^ (2021)	6.7 M	43.33	0.9917	1.8091	0.1756	37.60	0.9668	3.5959	0.7903
MC-CDic^8^ (2023)	9.6 M	43.55	0.9919	1.6970	0.1671	37.94	0.9700	3.4874	0.7612
DCAMSR^31^ (2023)	9.3 M	43.79	0.9920	1.6521	0.1654	37.98	0.9702	3.4751	0.7617
SANet^29^ (2024)	7.3 M	42.99	0.9914	1.8702	0.1801	37.15	0.9630	3.8740	0.8958
A2-CDic^27^ (2025)	10.1 M	44.10	0.9925	1.5939	0.1561	38.79	0.9720	3.2032	0.6769
**WCCAN (proposed)**	**5.2 M**	**44.20**	**0.9926**	**1.5750**	**0.1545**	**38.87**	**0.9721**	**3.1819**	**0.6710**

Bold: optimal outcome; underline: second-optimal outcome. The upward and downward arrows indicate that higher and lower values are better, respectively.

A2-CDic, alignment-assisted multi-contrast convolutional dictionary; HFEN, high-frequency error norm; MCMSR; multi-contrast MRI super-resolution; Params, parameters; PSNR, peak SNR; RMSE, root mean squared error; SCMSR, single-contrast MRI super-resolution; SR, super-resolution; SSIM, structural similarity index measure.

**Table 3:** Quantitative results of different SCMSR and MCMSR methods on M4Raw datasets under ×2 and ×4 scale factors

SR methods	Params	2×				4×			
		PSNR↑	SSIM↑	RMSE↓	HFEN↓	PSNR↑	SSIM↑	RMSE↓	HFEN↓
CSN^18^ (2019)	10.6 M	32.16	0.8159	6.4186	0.4121	29.10	0.7056	8.9806	1.6666
SRDenseNet^12^ (2022)	6.6 M	32.05	0.8161	6.3965	0.4206	29.06	0.7050	8.9866	1.6962
MRDN^11^ (2021)	8.7 M	32.22	0.8186	6.2742	0.4009	29.16	0.7084	8.9347	1.6083
EDSR + MHCA^19^ (2023)	2.5 M	32.29	0.8202	6.2212	0.3919	29.23	0.7098	8.8578	1.6040
**WCCAN (Baseline)**	3.5 M	**32.34**	**0.8204**	**6.1756**	**0.3875**	**29.50**	**0.7134**	**8.5910**	**1.4919**
MINet^10^ (2021)	6.7 M	32.55	0.8225	6.0314	0.3606	29.68	0.7212	8.4157	1.4469
MC-CDic^8^ (2023)	9.6 M	32.73	0.8252	5.9196	0.3551	29.90	0.7264	8.2061	1.3751
DCAMSR^31^ (2023)	9.3 M	32.84	0.8267	5.8428	0.3386	30.03	0.7258	8.0946	1.3416
SANet^29^ (2024)	7.3 M	32.47	0.8222	6.1050	0.3827	29.59	0.7180	8.4979	1.4832
A2-CDic^27^ (2025)	10.1 M	32.92	0.8273	5.7828	0.3360	30.11	0.7318	8.0193	1.3183
**WCCAN (proposed)**	**5.2 M**	**32.97**	**0.8276**	**5.7514**	**0.3225**	**30.26**	**0.7341**	**7.8934**	**1.2629**

Bold: optimal outcome; underline: second-optimal outcome. The upward and downward arrows indicate that higher and lower values are better, respectively.

A2-CDic, alignment-assisted multi-contrast convolutional dictionary; HFEN, high-frequency error norm; MCMSR; multi-contrast MRI super-resolution; Params, parameters; PSNR, peak SNR; RMSE, root mean squared error; SCMSR, single-contrast MRI super-resolution; SR, super-resolution; SSIM structural similarity index measure.

**Table 4:** Complexity analysis of different MCMSR methods with a resolution of 256 × 256 under a ×4 upscaling factor

Methods	Params (M)	FLOPs (G)	Peak GPU (MB)	Runtime (ms)
MINet^10^	6.7	284.88	3948.53	74.23
MC-CDic^8^	9.6	791.60	8268.81	69.87
DCAMSR^31^	9.3	131.67	3222.19	57.08
SANet^29^	7.3	324.69	4258.73	61.54
A2-CDic^27^	10.1	945.57	9839.13	103.11
**WCCAN**	**5.2**	**125.45**	**2974.93**	**51.16**

Bold values indicate the best performance for each metric.A2-CDic, alignment-assisted multi-contrast convolutional dictionary; DCAMSR, dual cross-attention multi-contrast super-resolution; FLOPs, floating-point operations; G, giga; M. mega; MB, megabyte; MC-CDic, multi-contrast convolutional dictionary; MCMSR, multi-contrast MRI super-resolution; MINet, multi-stage integration network; ms, milliseconds; Params, parameters; peak GPU, peak graphics processing unit; SANet, separable attention network; WCCAN, windowed cross-contrast attention network.

**Table 5:** Progressive ablation study of the proposed WCCAN components on the IXI and M4Raw datasets under ×4 scaling

Baseline	Ref image	WHPMB	TCCFM	SSAM	Params	IXI				M4Raw			
						PSNR↑	SSIM↑	HFEN↓	LPIPS↓	PSNR↑	SSIM↑	HFEN↓	LPIPS↓
**✓**	✗	✗	✗	✗	3.5 M	33.35	0.9278	1.4032	0.1108	29.50	0.7134	1.4919	0.3679
✓	✓	✓	✗	✗	3.7 M	36.09	0.9630	1.0022	0.0510	29.86	0.7257	1.3829	0.3512
✓	✓	✓	✓	✗	4.9 M	37.82	0.9679	0.7834	0.0356	30.02	0.7269	1.3261	0.3473
✓	✓	✓	✓	✓	5.2 M	**38.87**	**0.9721**	**0.6710**	**0.0325**	**30.26**	**0.7341**	**1.2629**	**0.3400**

The upward and downward arrows indicate that higher and lower values are better, respectively. Bold values indicate the best-performing variant.HFEN, high-frequency error norm; LPIPS, learned perceptual image patch similarity; M, mega; Params, parameters; PSNR, peak SNR; ref, reference; SSIM,structural similarity index measure; SR, super resolution; SSAM, selective spatial adaptive modulation; TCCFM, triple cross-contrast fusion module; WHPMB, wavelet-guided HF prior modeling block; WCCAN, windowed cross-contrast attention network.

**Table 6:** Quantitative assessment of model component contributions to SR performance and complexity on both IXI and M4Raw datasets under ×4 scale factor

Models	Params	IXI				M4Raw			
		PSNR↑	SSIM↑	HFEN↓	LPIPS↓	PSNR↑	SSIM↑	HFEN↓	LPIPS↓
Effectiveness of WHPMB									
Variant 1	4.8 M	37.29	0.9666	0.8913	0.0388	29.81	0.7221	1.3999	0.3495
Variant 2	5.3 M	38.41	0.9710	0.7291	0.0337	30.18	0.7329	1.2874	0.3417
Variant 3	5.2 M	**38.87**	**0.9721**	**0.6710**	**0.0325**	**30.26**	**0.7341**	**1.2629**	**0.3400**
Effectiveness of TCCFM									
Variant 1	4.0 M	37.69	0.9683	0.8037	0.0362	29.90	0.7257	1.3754	0.3481
Variant 2	5.2 M	**38.87**	**0.9721**	**0.6710**	**0.0325**	**30.26**	**0.7341**	**1.2629**	**0.3400**
Effectiveness of SSAM									
Variant 1	4.9 M	37.82	0.9679	0.7834	0.0356	30.02	0.7269	1.3261	0.3473
Variant 2	5.0 M	38.17	0.9714	0.7363	0.0341	30.09	0.7287	1.3183	0.3439
Variant 3	5.2 M	**38.87**	**0.9721**	**0.6710**	**0.0325**	**30.26**	**0.7341**	**1.2629**	**0.3400**

The upward and downward arrows indicate that higher and lower values are better, respectively. Bold values indicate the best-performing variant.HFEN, high-frequency error norm; LPIPS, learned perceptual image patch similarity; M, mega; Params, parameters; PSNR, peak SNR; SSIM,structural similarity index measure; SR, super resolution; SSAM, selective spatial adaptive modulation; TCCFM, triple cross-contrast fusion module; WHPMB, wavelet-guided HF prior modeling block.

## Supplementary Materials

### Supplemental Mathematical Formulations

***Wavelet-Guided High-Frequency Prior Modeling Block (WHPMB)***

The proposed WHPMB first splits the input features *f_Ref_* into two channel-wise branches *f*_1_ and *f*_2_.

In the first branch, Haar wavelet decomposition is applied to separate the features into low-frequency sub-band *f*_LL_ and high-frequency sub-bands*f_LH_*, *f_HL_*, and *f_HH_*:

(S1) $$f_{LL},f_{LH},f_{HL},f_{HH}=\text{DWT(}f_{1})$$

The low-frequency component is refined using a ConvBlock that consists of two 3×3 convolution layers followed by a GELU activation:

(S2) $$f_{LL}^{enh}=\text{ConvBloack}(f_{LL})$$

While the concatenated HF sub-bands are refined and enhanced using tanh activation function:

(S3) $$f_{HF}^{enh}=\;\tanh\;\left(\text{ConvBlock}\left(\left[f_{LH},f_{HL},f_{HH}\right]\right)\right)$$

The enhanced HF coefficients $f_{HF}^{enh}$, $f_{LH}^{enh}$, $f_{HL}^{enh}$, and $f_{HH}^{enh}$ and combined with $f_{LL}^{enh}$ via inverse DWT to reconstruct the enhanced $f_{1}^{enh}$ features:

(S4) $$f_{1}^{enh}=\text{ IDWT}\left(f_{LL}^{enh},f_{LH}^{enh},f_{HL}^{enh},f_{HH}^{enh}\right)$$

In the second branch, features *f*_2_ are refined using ConvBlock, and both branches are fused using a channel-wise gating mechanism (CWGM):

(S5) $$f_{cat}^{enh}=\;\sigma \left(FC_{2}\left(\text{ReLU}\left(FC_{1}\left(\text{GAP}\left(f_{cat}\right)\right)\right)\right)\right)$$

Where σ(.) is the sigmoid activation, GAP(.) denotes global average pooling, FC1 and FC2 are fully connected layers. The final output feature is obtained as:

(S6) $$f_{Gated}=f_{cat}^{enh}\cdot f_{cat}$$

Where ⊙ denotes element-wise multiplication.

***Windowed Cross-Contrast Attention Module(WCCAM)***

The proposed WCCAM computes window-based cross-attention as:

(S7) $$Att_{Win}=\text{Softmax}\left[\frac{Q_{Win}K_{Win}^{T}}{\sqrt{C}}+B\right]V_{Win}$$

Where AttWin represents the attended features, *Q_win_*, *K_win_* and *V_win_* are the windowed query, key, and value features respectively, and *B* denotes the learnable relative positional encoding used in ^43,45^.
